# Prevalence and risk factors associated with precancerous and cancerous cervical lesions among HIV-infected women in University of Gondar specialized comprehensive referral hospital, Northwest Ethiopia: cross-sectional study design

**DOI:** 10.1186/s12905-024-03174-0

**Published:** 2024-06-04

**Authors:** Elfalet Worku, Getachew Yigizaw, Robel Admassu, Dawit Mekonnen, Winta Gessessa, Zemenu Tessema, Tarkie Walle

**Affiliations:** 1 Gynecology oncologist Department of Obstetrics and Gynecology College of Medicine and Health Sciences, University of Gondar, Gondar, Ethiopia; 2 Departments of Epidemiology and Biostatistics, Institute of Public Health, College of Medicine and Health Sciences, University of Gondar, Gondar, Ethiopia; 3 Department of Surgical Nursing, School of Nursing, College of Medicine and Health Sciences, University of Gondar, Gondar, Ethiopia

**Keywords:** Cancer, Cervix, Precancerous, HIV positive

## Abstract

**Background:**

Cervical cancer is one of the leading causes of death in women worldwide. The majority of the cases are found in developing countries. The increasing risk of cervical cancer prevalence and growing danger of death from cervical cancer and the high occurrence of human papillomavirus (HPV) infection in women who are HIV positive give us the ground to study the prevalence and associated risk factors.

**Objective:**

The study aims to assess the prevalence of cervical cancer screening and associated risk factors among HIV-positive women attending the Adult ART clinic at the University of Gondar Hospital.

**Methods:**

An institution-based cross-sectional study was conducted from March to August 2021, on adult HIV-positive women attending the Adult ART clinic at Gondar University Referral Hospital by phone calling patients per week for six months to complete a total of 2744 HIV-positive patients who were not screened for cervical cancer before. The data were collected using an interviewer-administered questionnaire. Bivariate and multivariable logistic regression analyses were used to determine the presence and the degree of association between dependent and independent variables. In the multivariable logistic analysis, a P-value of < 0.05 and odds ratio with a 95% confidence interval were considered to determine independent predictors for the prevalence of premalignant or malignant cervical lesions among HIV-positive patients.

**Result:**

This study assessed 915 HIV Positive women who were screened for cervical cancer via visual inspection with acetic acid (VIA) as the primary screening tool and found that 24.48% had positive VIA results. Those with VIA-positive cases pathology examination showed 72.4% had abnormal pathology reports (CIN 1/2/3–51.25%, 17.23% cancer & 3.9% CIS), strengthening the finding in many studies that suggest HIV-positive women have a high rate of premalignant lesions.

## Introduction

Cervical cancer ranks as the second most prevalent form of cancer and represents the leading cause of cancer-related deaths among women on a global scale. It has been firmly established that human papillomavirus (HPV) infection is a significant contributing factor to the development of cervical cancer. Moreover, mounting evidence links HPV to other types of anogenital cancers (such as anus, vulva, vagina, and penis) as well as head and neck cancers [1–3].

Cervical cancer is a major public health issue that affects women in both developed and developing nations. The burden of cervical cancer is highest in Sub-Saharan Africa, especially in Eastern African countries, where it accounts for approximately 23.3% of morbidity and 16.54% of mortality. The overall burden of cervical cancer is expected to increase further. Cervical cancer is the third most common cancer among women globally, with a range of 2.4 to 25.2%, and the second most common cancer in Africa, with a rate of 25.2% [3–5].

In 2012, there were an estimated 266,000 deaths and 528,000 new cases of cervical cancer; approximately 85% of these cases and deaths happened in less-developed areas [5]. Cervical cancer (CC) is the most common cancer among women in sub-Saharan Africa and the fourth most common cancer in the world [5]. The primary cause of cervical cancer is ongoing infection with specific strains of the human papillomavirus (HPV). Approximately 70% of cervical cancer cases worldwide are caused by HPV types 16 and 18 [6]. Additional risk factors include smoking, having multiple sexual partners, and starting sexual activity before the age of 20 [7, 8]. Both the incidence and mortality of cervical cancer have increased, placing a significant financial and social burden on society. Fortunately, HPV 16 and 18-specific vaccinations are now readily available and are intended to prevent infection [1, 4].

Human papillomavirus (HPV) and HIV infection are highly prevalent in Sub-Saharan Africa [6, 7]. Increased risk of cervical cancer, decreased clearance of precancerous lesions and HPV, and higher rates of HPV acquisition are all linked to HIV [8–10, and 11]. There is an increasing need for cervical cancer prevention, especially in low-resource settings, as HIV-positive women receive antiretroviral therapy (ART) [12, 13]. Despite continuous efforts to increase screening, HPV vaccinations and cervical cancer screening are very scarce in developing countries. In the last three years, less than 6% of eligible women in Kenya had a cervical cancer screening, compared to 23% in South Africa [9, 14, and 15].

After breast cancer, cervical cancer is the second most common cause of cancer in Ethiopia and the leading cause of death for cancer patients who are female [15]. Before the Single Visit Approach (SVA) for CCP service was introduced by Pathfinder International in 2009, the majority of Ethiopian women had extremely limited access to cervical cancer screening. Ethiopia has an estimated age-standardized incidence rate of 26.4/100,000 women for cervical cancer, based on a 2012 estimate [3, 6, 9].

HIV infection has been linked to an increased occurrence of HPV infection and a decreased ability to clear it. HIV-positive women have a higher likelihood of acquiring HPV infection compared to HIV-negative women, particularly those with low CD4 counts and high HIV viral load [2, 15]. According to the 2021 guidelines from the World Health Organization (WHO), in situations where HPV DNA screening is not feasible, regular cervical cancer screening using visual inspection with acetic acid (VIA) or cytology every 3 years can be employed as the primary screening method for both HIV-negative and HIV-positive women [16–18].

The Ethiopian Ministry of Health’s 2021 cervical cancer guideline recommended that cervical cancer screening be included in routine HIV/AIDS care and that all women living with HIV/AIDS should be screened for cervical cancer at the time of diagnosis and every two years after that using VIA or cytology [19–21]. This is because women living with HIV/AIDS are more likely to develop cervical cancer. Even though it was intended to screen all women living with HIV/AIDS, only 16% of HIV-positive women were screened for cervical cancer, according to EPHIA (Ethiopian Population-based HIV Impact Assessment) 2020 reports [22, 23].

The University of Gondar Comprehensive Referral Hospital, a prominent teaching hospital in the Amhara region, provides adult antiretroviral therapy (ART) services. However, a study conducted in 2016 involving 496 HIV patients revealed a low participation rate in cervical cancer screening within the institution [9]. The forthcoming study aims to investigate the prevalence and associated risks of precancerous and cancerous cervical lesions among HIV-infected women who attend the Adult ART clinic at the University of Gondar referral hospital and have not undergone cervical cancer screening. Notably, this study stands out due to its inclusion of a substantial sample size of 915 HIV-positive patients, the largest number considered for such research.

### Justification of the study

In 2008, one in every six cancers worldwide was caused by an infection that could be prevented or treated [5]. Cervical cancer is the only gynecologic cancer for which a screening test is available that can detect its pre-cancerous stage. The World Health Organization recommended that cervical cancer screening is needed for women aged 30–49 years irrespective of their HIV status. It is also recommended that sexually active girls and women need to be screened as soon as they are diagnosed as positive for HIV. Cervical cancer disproportionately affects women in low- and middle-income countries (LMICs) where it is the second most common cancer in women. LMICs account for 84% of new cases worldwide with 8 out of 10 cases occurring in Sub-Saharan Africa [12].

In Ethiopia, visual inspection with acetic acid (VIA) as a screening tool and Cryotherapy as a treatment modality for premalignant cervical lesions are available in some hospitals. The service is mainly for HIV-positive women. However, there is limited information about the level of cervical cancer screening service utilization in Ethiopia in general and in the study area in particular. Therefore, this study aimed to assess the prevalence of cervical Cancer screening and associated factors among HIV-positive women.

Therefore, this study may assist policymakers in developing guidelines for prevention and treatment strategies for cervical cancer among HIV-infected women which is largely based on limited evidence taken from prior studies. Thus, it is expected to help policy/decision makers, non-government organizations (NGOs), health care providers, and community service providers design strategies and take necessary interventions accordingly.

## Objectives

### General objective

To assess the prevalence and associated risk factors for precancerous cervical lesions in HIV-infected women in Gondar University referral hospital, Northwest Ethiopia.

### Specific objectives

To assess the prevalence of precancerous cervical lesions in HIV-infected women in Gondar University referral hospital, Northwest Ethiopia.

To assess associated risk factors of precancerous cervical lesions in HIV-infected women in Gondar University referral hospital, Northwest Ethiopia.

## Methodology

### Study design and period

An institution-based cross-sectional study was conducted among HIV-positive women attending adult ART (Anti-Retroviral Therapy) and referred to the cervical cancer screening Clinic at Gondar University outpatient department from Referral Hospital, northwest Ethiopia. The study was conducted from March to August 2021.

### Description of study area

The study was conducted at the University Gondar Comprehensive Referral Hospital, one of the specialized Tertiary Teaching Hospitals; which is among the pioneer hospitals in the country serving the population for more than the last 60 years. It is located in North West Ethiopia, in the Amhara Region; 741 km away from Addis Ababa i.e., the capital city of Ethiopia.

It is one of the teaching hospitals giving more than 10 undergraduate postgraduate, and subspecialty programs in medicine and other health-related sciences. Department of Obstetrics and Gynecology started a specialty program in 2005. Currently having more than 50 residents; recently also opened sub-specialty programs in three fields thus maternal-fetal medicine is one.

The HIV care service of the hospital was established in 2013 and has three clinics: Adult ART, pediatric ART, and Volunteer Counseling and Testing (VCT). There were about 9565 HIV-positive clients enrolled in the adult ART Clinic. From these, 4731 clients were on Highly Active Antiretroviral Therapy (HAART). Before the emergence of visual inspection with acetic acid (VIA) for cervical Ca screening, a Pap smear was used. However, it was not routinely used for all HIV-positive women. Instead, the physician ordered the Pap smear only for women who were suspected of or have symptoms of cervical Ca irrespective of their HIV serostatus. Premalignant cervical cancer screening by visual inspection with acetic acid (VIA) and cryotherapy services have been available in the Hospital since 2013. In principle, the screening service for pre-cancerous cervical lesions using VIA has to be given routinely to all HIV-positive women free of charge, and the screening service can be repeated every three years. However, what is being done is that providers selectively order/direct some women to screen. Trained nurses and physicians, who are assigned in the ART clinic, have the responsibility to provide information, education, and counseling (IEC) and the screening service.

In our study, we assessed the prevalence and associated risk factors for 915 HIV-positive patients at the Adult ART clinic who were not screened for cervical cancer due to different reasons before. The screening was carried out by the Gynecology Oncology team (Fellows & Gynecology Oncologists) in collaboration with midwives & nurses at Gondar University Hospital gynecology outpatient in a cervical cancer screening room five days a week from March to August 2021.

### Source population

All women who come for cervical cancer screening at Gondar University outpatient department from Referral Hospital, northwest Ethiopia,

### Study population

All HIV-positive women aged 18 years and above who were receiving care in the Adult ART Clinic during the study period were the study population.

### Inclusion and exclusion criteria

#### Inclusion criteria

All HIV-positive women aged 18 years and above who were receiving care in the Adult ART clinic during the study period were the study population.

#### Exclusion criteria

HIV Positive patients with acute life-threatening illness were not included in the study.

#### Sampling method & sample size determination

2744 HIV-positive patients are having followed up at Gondar University Hospital who were not screened for cervical cancer. An institution-based cross-sectional study was conducted from March to August 2021, on adult HIV-positive women attending the Adult ART clinic at Gondar University Referral Hospital by phone calling 38 patients per week for 6 months to complete a total of 915HIV-positive patients that were not screened for cervical cancer before.

#### Data collection technique

The questionnaire was prepared in English and translated into Amharic. The local language, and then retranslated to English by language experts. Four first-degree graduate nurses were employed for data collection. One day before training was given to data collectors on the methods, objectives, and tools before collection began. The tool was pre-tested on 20 cases to see the accuracy of responses, the clarity of language, and the appropriateness of the tool.

#### Data analysis and interpretation

The collected data were checked and coded manually entered into Epi-info version 7 and then exported to Statistical Package for Social Sciences (SPSS) version 20.0 for statistical analysis. Descriptive statistics and binary logistic regression analyses were done. In the bivariate analysis, variables that had a significant association with the outcome variable at 0.2 p-values were considered for multivariable analysis. The Backward Wald method was employed in the multivariable analysis. In the multivariable logistic regression analysis, p-value less than 0.05 and adjusted odds ratio with a 95% confidence interval were used to determine the presence and degree of association between dependent and independent variables. The model fitness test was checked using Hosmer and Lemeshow statistics (*P* = 0.71). The Odds ratio with the corresponding 95% confidence interval was calculated to show the strength of the association.

### Operational definition

#### Uptake of cervical cancer screening

HIV-positive women who were screened for a premalignant cervical lesion at least once in their lifetime (self-reported).

#### Awareness of cervical cancer screening

HIV-positive women who had heard about cervical cancer and cervical CA screening.

## Result

### Socio-demographic characteristics

A total of 915 women living with HIV were included in this study. The median age of women was 36 years with IQR 8. The majority of women aged 592(64.70%) were 35 and above. A total of 834(91.15%) women living with HIV had no formal education. The majority of women 802(87.65%) were urban residents. A total of 870 (95.08%) women living with HIV had good drug adherence. A total of women 445(48.63%) had a history of transmitted disease and 771(84.26%) of them were HIV WHO stage I (Table 1).

**Table 1:** Socio-demographic and clinical characteristics of respondents among women who live with HIV

Variables	Category	Frequency (n = 915)	Percentage
Visual inspection with acetic acid(VIA) test result	Negative	691	75.52
	Positive	224	24.48
Age group	15–24	28	3.06
	25–34	295	32.24
	35+	592	64.70
Marital status	Single	496	54.21
	Married	419	45.79
Residence	Urban	802	87.65
	Rural	113	12.35
Educational status	No education	834	91.15
	Primary	41	4.48
	Secondary and above	40	4.37
Number of children	< 2	724	79.13
	>=2	191	20.87
Drug adherence	Good	870	95.08
	Poor	45	4.92
VIA treatment	No treatment	691	75.52
	cry therapy	6	0.66
	Thermo coagulation	19	2.08
	LEEP	61	6.67
	Punch biopsy	138	15.08
Follow up	3–6 month	262	28.63
	1–3 year	653	71.37
Visit type	First	904	98.80
	Second(rescreening)	11	1.20
Sexually transmitted disease	Yes	445	48.63
	No	470	51.7
Initiation of ART	Yes	884	96.61
	No	31	3.39
HIV WHO stage	Stage I	771	84.26
	Stage II	40	4.37
	Stage III	16	1.75
	Stage IV	88	9.62
Pathology	Not collected/not done	768	83.83
	Negative	41	4.48
	CIN (1,2,3)	76	8.31
	Carcinoma in situ	5	0.55
	Invasive cervical cancer	25	2.75

### Prevalence of precancerous lesions of the cervix among women living with HIV

**Fig. 1:**
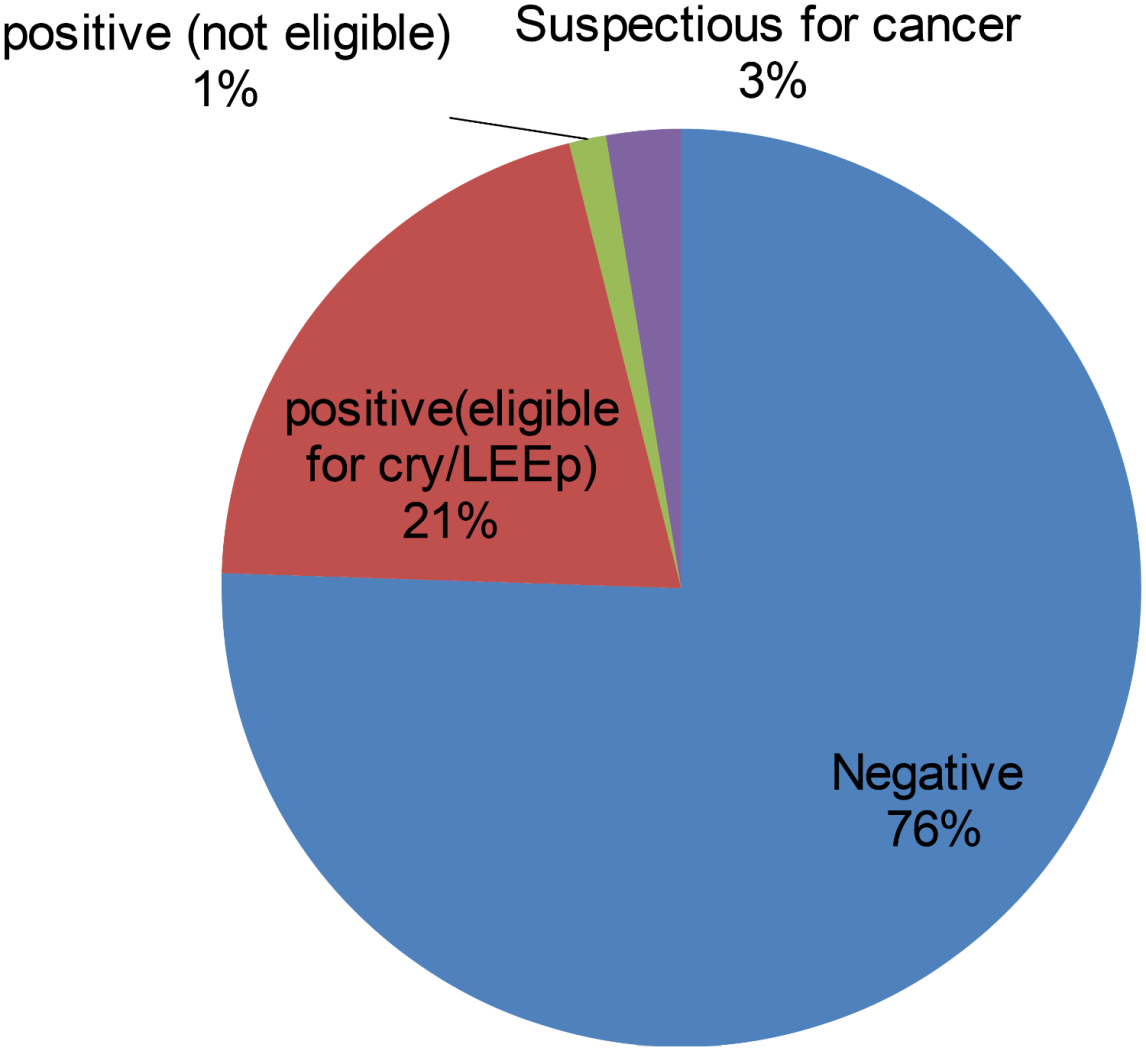
Visual inspection of the cervix with acetic acid (VIA) test result among women living with HIV who had follow-ups at gondar comprehensive specialized hospital

The prevalence of precancerous lesions of the cervix among women living with HIV who had follow-ups at Gondar Comprehensive Specialized Hospital was 24.48% with a 95% confidence interval of 21.79–27.37%. The VIA test result showed that a total of 224 women showed a positive test result. The positive result had three levels that 188(20.55%) are eligible for cry/LEEP, 12(1.31%) are not eligible for cry/LEEP and 24(2.62%) of women are suspicious for cancer (Fig. 1).

### Factors associated with precancerous lesions of the cervix among women living with HIV

In the bi-variable analysis result, age group, marital status, residence, educational status, number of children, ART drug adherence, sexually transmitted disease, ART initiation, and HIV WHO stage were factors associated with precancerous lesions of cervical cancer. In the multivariable logistic regression model:- residence, ART drug adherence, having sexually transmitted disease, ART initiation, and HIV WHO stage were statistically significant factors associated with precancerous lesions of cervical cancer among women living with HIV who had follow-up at Gondar Comprehensive Specialized Hospital.

The odds of developing precancerous cervical lesions among women living in urban were decreased by 62% (AOR: 0.38, 95%CI: 0.24, 0.62) as compared to women living in rural. The odds of developing precancerous cervical lesions among women who had poor ART drug adherence were three (AOR: 3.02, 95%CI: 2.03, 6.25) times higher as compared to women with good ART drug Adherence. The odds of developing precancerous cervical lesions among women who had no sexually transmitted disease were decreased by 36% (AOR: 0.64, 95%CI: 0.46, 0.89) as compared to women who had STI. The odds of developing precancerous cervical lesions among women who had no initiated ART were four (AOR: 4.02, 95%CI: 1.05, 17.81) times higher as compared to women who had initiated ART. The odds of developing precancerous cervical lesions among women whose HIV WHO stage IV were five (AOR: 5.46, 95%CI: 3.29, 9.0) times higher as compared to women whose HIV WHO stage I (Table 2).

**Table 2:** Bi-variable and multivariable logistic regression model for VIA test results among women living with HIV who had follow-ups at Gondar comprehensive specialized hospital

Variables	category	COR(95%CI)	AOR(95%CI)
Age group	15–24	1	1
	25–34	1.17(0.48,2.86)	1.04(0.40,2.68)
	35+	0.87(0.36,2.10)	0.71(0.28,1.79)
Marital status	Single	1	1
	Married	0.68(0.50,0.93)	1.03(0.72,1.47)
Residence	Rural	1	1
	Urban	0.36(0.24,0.54)	0.38(0.24,0.62)**
Educational status	No education	1	1
	Primary	0.60(0.26,1.38)	0.83(0.38,2.01)
	Secondary and above	0.41(0.16,1.08)	0.53(0.19,1.44)
Number of children	< 2	1	1
	>=2	0.71(0.48,1.06)	0.61(0.39,1.05)
Drug adherence	Good	1	1
	Poor	5.12(2.76,9.49)	3.02(2.03,6.25)*
Sexually transmitted disease	Yes	1	1
	No	0.52(0.38,0.71)	0.64(0.46,0.89)**
Initiation of ART	Yes	1	1
	No	5.24(2.52,10.89)	4.18(1.25,17.81)*
HIV WHO stage	Stage I	1	1
	Stage II	2.78(1.44,5.37)	1.33(0.54,3.28)
	Stage III	3.24(1.18,8.85)	2.56(0.82,7.97)
	Stage IV	6.02(3.80,9.56)	5.46(3.29,9.07)***

*= significant at alpha 0.05, **=significant at alpha 0.01, ***=significant at alpha 0.001

## Discussion

Of the global burden of cervical cancer, 85% is found in sub-Saharan countries where awareness about cervical cancer prevention and screening practices is low. The region has a high prevalence of HPV infection which is ascribed as a cause of cervical cancer in almost 99% [6, 7]. HIV-positive women have a higher incidence of persistent HPV infection which leads to an increased risk of developing premalignant lesions of the cervix. The risk of developing invasive Cervical Ca in HIV-positive women is ten years earlier than HIV HIV-negative women [1, 4].

Several studies have revealed a notable prevalence of precancerous and cancerous cervical lesions (PCCL) in developing countries like Ethiopia, highlighting the significance of cervical cancer and HIV infection as major health concerns for women [2, 3, 19]. The objective of this particular study was to examine the occurrence and risk factors associated with PCCL among HIV-infected women at the University of Gondar Specialized Comprehensive Referral Hospital in Northwest Ethiopia. In a prior cross-sectional study conducted at Gondar University Hospital, it was discovered that HIV-positive women had a remarkably low rate of cervical cancer screening, specifically 10% [9]. Expanding on this previous research, our study focused on examining the presence of precancerous and cancerous cervical lesions among women living with HIV/AIDS who primarily underwent cervical cancer screening using VIA (Visual Inspection with Acetic Acid).

The findings of this study revealed that the prevalence of precancerous and cancerous cervical lesions among HIV-positive women was 24.48%, with a 95% confidence interval ranging from 21.79 to 27.37%. This figure is similar to the results of a study conducted in Tanzania [24], which reported rates of 26.8%, and another study in Kenya [25] with rates of 26.7%. Though, in comparison to other research conducted in Uganda [26], Cote d’Ivoire [27], Cameroon [28], and southern Ethiopia [29], the prevalence of precancerous and cancerous cervical lesions in this study was higher, with rates of 3%, 9%, 12.2%, and 18.7% respectively. The observed variations in the prevalence of precancerous and cancerous cervical lesions (PCCL) between this study and previous research could be potentially attributed to differences in participant socio-demographic characteristics and the specific study population. It is important to note that most of the previous studies encompassed both HIV-positive and HIV-negative individuals, whereas the present study exclusively focused on HIV patients. By narrowing the study population solely to HIV-positive individuals, it is possible that the higher proportion of PCCL cases found in this study can be attributed to the increased vulnerability of this specific group. HIV-positive individuals are known to be at a higher risk of developing PCCL due to factors such as compromised immune function and a higher prevalence of persistent HPV infection. The higher prevalence of precancerous and cancerous cervical lesions (PCCL) identified in this study underscores the urgent need for expanded screening services throughout healthcare facilities in the region. The findings highlight the importance of reaching out to vulnerable communities and providing them with comprehensive health education regarding cervical cancer and its screening.

In our study among HIV/AIDS-positive that were VIA positive, 72.4% had abnormal pathology reports (CIN 1/2/3–51.25%, 17.23% cancer & 3.9% CIS), strengthening the finding in many studies that suggest HIV-positive women have a high rate of premalignant lesions. Therefore, HIV-positive women may be at risk for overtreatment in screening programs as they have a high prevalence of low-grade lesions that infrequently progress to HSIL.To avoid the early intervention of low-grade lesions detected in HIV patients, the United States changed screening guidelines for HIV patients from annual to every 3 years consecutive normal screens [20, 21]. This is true for low and middle-income countries like Ethiopia where the “See and Treat Approach” with single-visit diagnosis and treatment is practiced [19, 30].

Based on a systematic review and meta-analysis conducted in the United States in 2018, it was found that women with a low CD4 count and high HIV viral load had a heightened likelihood of acquiring HPV. However, the risk was reduced among women who adhered to antiretroviral therapy (ART). Similarly, in our study, we observed that women who were at WHO stage 4 of HIV and those who did not initiate ART drugs at the time of diagnosis had a 5-fold and 4-fold increased risk, respectively, of developing precancerous and cancerous conditions in the cervix. Poor adherence to ART, as self-reported or retrieved from medical records, was associated with a 3-fold increased risk of having abnormal screening results compared to those with good adherence to ART [28, 29, 31, 32].

The likelihood of developing precancerous cervical lesions was 36% lower in women without any sexually transmitted diseases compared to those with an STI. Additionally, the odds of developing these lesions were reduced by 62% in women living in urban areas compared to those living in rural areas. Often, patients with sexually transmitted diseases have a history of multiple sexual partners, which increases their exposure to HPV, a primary cause of precancerous cervical lesions. On the other hand, urban residents may have a better awareness of the importance of early screening and treatment, supported by various studies conducted in different areas [26, 28, 30].

## Conclusion

This study assessed 915 HIV Positive women who were screened for cervical cancer via visual inspection with acetic acid (VIA) as the primary screening tool and found that 24.48% had positive VIA results. Those with VIA-positive cases pathology examination showed 72.4% had abnormal pathology reports (CIN 1/2/3–51.25%, 17.23% cancer & 3.9% CIS), strengthening the finding in many studies that suggest HIV-positive women have a high rate of premalignant lesions. The study also found a significant association with WHO Stage 4 at the time of diagnosis, no initiation of ART, and self-reported poor adherence to ART as an associated factor for abnormal cervical cancer results. Based on these findings, there is a need to expand screening services by establishing accessible and well-equipped facilities that can provide regular screenings to women at risk. By increasing the availability of screening services, more women can be reached and diagnosed at earlier stages, improving their chances of successful treatment and reducing the burden of advanced cervical cancer cases.

## Data Availability

All relevant data and materials included in the manuscript.
